# Male resource defense mating system in primates? An experimental test in wild capuchin monkeys

**DOI:** 10.1371/journal.pone.0197020

**Published:** 2018-05-22

**Authors:** Barbara Tiddi, Michael Heistermann, Martin K. Fahy, Brandon C. Wheeler

**Affiliations:** 1 Cognitive Ethology Laboratory, German Primate Center, Göttingen, Germany; 2 Department of Behavioral Ecology, Johann-Friedrich-Blumenbach Institute for Zoology and Anthropology, Georg-August Universität Göttingen, Göttingen, Germany; 3 Endocrinology Laboratory, German Primate Center, Göttingen, Germany; 4 CERCOPAN Nigeria, Calabar, Cross River State, Nigeria; 5 School of Anthropology & Conservation, University of Kent, Canterbury, United Kingdom; University of Texas at El Paso, UNITED STATES

## Abstract

Ecological models of mating systems provide a theoretical framework to predict the effect of the defendability of both breeding resources and mating partners on mating patterns. In resource-based mating systems, male control over breeding resources is tightly linked to female mating preference. To date, few field studies have experimentally investigated the relationship between male resource control and female mating preference in mammals due to difficulties in manipulating ecological factors (e.g., food contestability). We tested the within-group male resource defense hypothesis experimentally in a wild population of black capuchin monkeys (*Sapajus nigritus*) in Iguazú National Park, Argentina. *Sapajus* spp. represent an ideal study model as, in contrast to most primates, they have been previously argued to be characterized by female mate choice and a resource-based mating system in which within-group resource monopolization by high-ranking males drives female mating preference for those males. Here, we examined whether females (N = 12) showed a weaker preference for alpha males during mating seasons in which food distribution was experimentally manipulated to be less defendable relative to those in which it was highly defendable. Results did not support the within-group male resource defense hypothesis, as female sexual preferences for alpha males did not vary based on food defendability. We discuss possible reasons for our results, including the possibility of other direct and indirect benefits females receive in exercising mate choice, the potential lack of tolerance over food directed towards females by alpha males, and phylogenetic constraints.

## Introduction

Ecological models of mating systems explain the variety of mating systems across animals by considering the influence of ecological factors on the distribution of receptive females and the effect of the spatio-temporal distribution of receptive females on shaping male mating strategies [1–3]. As such, these models allow for predictions to be made regarding the organization of mating systems by considering the economic defendability of both breeding resources and mating partners.

Within this framework, resource-based mating systems are seen in cases in which males are able to control breeding resources that are essential for females [1,2]. Resource-based mating systems are classically assumed to occur when breeding resources (e.g., nesting sites, nuptial gifts, feeding territories) are economically defendable and males can competitively exclude others from them. In these cases, asymmetry in competitive abilities among males is assumed to determine male success in controlling resources [4] and, ultimately, mating advantage via female choice [5]. Key to resource-based mating systems is the idea that the greater a male’s ability to defend an important resource from other males is, the higher is his probability of attracting females [6,7]. Recently, empirical and experimental studies have shown that differences across males in the ability to control access to resources are a salient component of many resource-based mating systems, since this differential ability strongly influences mechanisms of female mate choice in a number of animal species [8–13].

When females are philopatric and live in stable groups, single or multiple males may in some cases defend the resources in the females’ home range [1]. These resource-based mating systems slightly depart from the classic idea of solitary females associating with individual males defending different territories, because multiple group males contribute to defend territories of resident females from other groups (cooperative male resource defense polygyny) [3]. Likewise, if resource defense is a male mating strategy, then variation in male ability to control within-group access to resources may affect female mate choice among group males in a similar way, with females preferring those males that overall are better able to defend and provide access to resources in within-group competition.

Although there is little evidence of such within-group resource defense mating systems in primates or other taxa (see [3] for a recent review of primate mating systems), robust capuchin monkeys (*Sapajus* spp.) have been argued to present such a case. Specifically, Janson [14,15] noted that the top ranking male (hereafter alpha male) in robust capuchins tends to secure the greatest access to monopolizable resources via aggressive competition, resulting in a high resource holding potential that allows him to differentially distribute access to the contestable feeding resources across females and/or their offspring. Further, females in these species actively solicit particular males and heavily bias their solicitations towards the alpha male ([16,17] reviewed in [18]). This scenario contrasted with observations of the closely-related white fronted capuchins (*Cebus albifrons*), wherein alpha males appear both less able to control access to feeding resources and to be less preferred by females [19]. Thus, in line with Emlen & Oring's [2] original idea of resource defense polygyny wherein a single resource-controlling male attracts several females, Janson [19] argued that female mating preference for alpha males among robust capuchins may be largely driven by their within-group resource monopolization. Further, the link between feeding ecology and robust capuchin mating systems is indicated by the fact that between-species variation in female preference for the alpha male in the genus *Sapajus* appears to be associated with within-group variation in the ability of males to control access to resources [20]. As such, male food monopolization may have a critical influence on female mate choice and explain the highly skewed mating pattern observed in the species. Yet, because of the difficulties imposed by natural settings (e.g., little control of food patch size, distribution and abundance) [21], no study has thus far experimentally tested the degree to which alpha male resource control is truly linked to female preference for this male, and thus whether within-group resource defense is a male mating strategy in robust capuchin monkeys.

In this study, we experimentally induced variation in the potential for alpha males to defend resources in two wild groups of black capuchin monkeys (*Sapajus nigritus*). We did so by manipulating the distribution of a preferred food (fruit) to test whether such manipulations induce changes in female preferences for the alpha male. Specifically, in each group we tested female preference under a clumped food condition in which alpha males could easily monopolize an entire food patch, and a dispersed condition in which alpha males could not effectively exclude other individuals from feeding [15]. We predicted that if resource defense is a male mating strategy, then female mating preferences for alpha males are contingent upon the latter’s ability to control access to high-quality resources. Therefore, we expect that females should direct a larger share of solicitations and copulations to the alpha male (i.e., be less promiscuous) during periods in which his potential to control access to resources is high (the clumped condition), relative to periods in which this potential is reduced (the dispersed condition). In addition, we explored both potential social aspects (e.g., whether females actually gain access to food controlled by males) and female features (reproductive and social parameters) that may influence the relationship between male resource control and female preference in this species.

## Materials and methods

### Study site and subjects

The study was conducted in Iguazú National Park, Argentina (25°, 40’S, 54°, 30’W), at the southwestern limit of the South American Atlantic Forest. This eco-region is characterized by humid, subtropical rainforest, where marked seasonality in daylight duration and temperature affects resource availability by drastically reducing the abundance of pulpy fruits and vertebrates during the austral winter from May to August [22]. Specifically, with pulpy fruit productivity dropping from 1000-1400g in the austral summer to 50-200g (dry weight/ha/day) in the austral winter, black capuchins face low food availability and depend mainly on dispersed fallback food in the latter season [23]. This naturally occurring variation in food availability allows researchers to experimentally introduce high value foods (e.g., bananas) during the winter months and thus exercise a great degree of control over the distribution and abundance of their most preferred foods [24].

Black capuchins are highly frugivorous primates endemic to this eco-region, and live in stable multimale-multifemale groups characterized by female philopatry and male dispersal, with males typically dispersing between 5 and 7 years of age [25]. Their social groups are characterized by despotic and linear within-group dominance relationships, with dominant individuals winning contests over food and preferred spatial positions [25,26]. Black capuchin females at the study site can be sexually receptive throughout the year, but show high seasonality in reproduction, with a mating season occurring mainly between May and late August [25].

The current study was conducted during two consecutive mating seasons (from early May to late August) in 2012 and 2013. We collected data on 12 sexually mature females belonging to two well-habituated groups, the Macuco group and the Spot group. The two groups comprised, respectively, 26 and 15 potentially sexually active sub-adult and adult individuals (i.e., females that have shown sexually proceptive behaviors, typically first occurring at 4.5 years of age, and non-natal males; see Table A in S1 File) at the beginning of the study (Macuco group: 7 females and 5 males; Spot group: 4 females and 3 males). As these groups have been subjected to long-term observations over the last two decades [25], all individuals were readily identified based on fur and facial patterns. Group members were arranged into a linear dominance hierarchy based on the direction of decided dyadic aggressive and approach-avoidance interactions using Matman 1.1 [27].

### Experimental protocol

We carried out controlled experiments with food provisioning platforms in order to manipulate food distribution during the austral winter months, and thus to take advantage of the period of low fruit availability. By doing so, we artificially altered the alpha males’ degree of control over high-value food resources within their groups. Specifically, we distributed the same quantity of high valuable food (12 bananas cut into 5–7 pieces each) on either one single provisioning platform (clumped condition with high potential resource control for alpha males) or on three different platforms (dispersed condition with relatively low potential resource control for alpha males). Each study group was presented with both a clumped and a dispersed condition, with each condition lasting for an entire 3.5-month field season (clumped for the Macuco group and dispersed for the Spot group during the entire winter season of 2012, and *vice versa* in the winter of 2013).

For each group, we selected three platform sites within the group home range with an inter-site distance of approximately 250-350m. Wooden platforms measuring approximately 1m x 1m were suspended from tree branches at a height of 3-10m above the ground by a system of ropes and pulleys (see [15,28]). Previous research at the field site has shown that alpha males effectively monopolize access to platforms, although within-site platform distance is a critical parameter that affects food monopolization, with alpha males being unable to monopolize multiple platforms that are widely spaced (>10m) [15]. When food is distributed across multiple platforms with such a degree of spacing, the relative ingestion rates of dominants decrease while other high-ranking group males are able to simultaneously feed from and control access to the additional platforms as successfully as the alpha male. We therefore optimized our low resource control condition by separating the three platforms from each other with an intra-site distance of 15-20m [15].

During the study periods, all platform sites were set up daily according to the specific conditions (low versus high resource control), so that both groups received the same experimental treatment up to three times per day (totaling 36 bananas/ day/group, which represents approximately half of the group’s daily energetic requirements). The importance of this introduced resource to the subjects is evinced by the relatively high rates of agonism that occur while feeding at the platforms relative to that which occurs when feeding on naturally-occurring resources, the fact that both groups typically visited all 3 sites within their home range daily, and that they normally began doing so soon after activity began in the morning (Tiddi & Wheeler, unpublished data). In order to minimize the effect of random arrivals of a subset of the group, platforms loaded with bananas were raised only once we made sure that a clear majority of the group was approaching the provisioning site (typically more than two-thirds of the group); platforms were not raised in cases in which too few individuals arrived or the alpha male did not show up at the site. Overall, we conducted a total of 440 provisioning events for the two groups over the two mating seasons, distributed uniformly across the four calendar months (Macuco group: 69 experimental events in the clumped and 148 in the dispersed resource control condition; Spot group: 93 experimental events in the clumped and 150 in the dispersed resource control condition). A concerted effort was made to evenly spread the experimental events for each group across the two conditions (clumped vs. dispersed); note, however, that the clumped resource setting indirectly influenced group daily visits to the platform sites by quickly satiating the alpha male and thus likely decreasing his motivation to direct the group to the next provisioning sites.

### Behavioral observations

Behavioral observations were conducted in both natural contexts and during feeding platform experiments. Groups were followed every day from dawn to dusk by two to three field assistants for each group, with an attempt made to monitor sexually proceptive females continuously. Females were identified as proceptive when showing conspicuous estrus-specific vocalizations [29], facial expressions (including eyebrow raising and grinning), and gestures (including chest rubbing and head cocking), typically while closely following a target male [16,17,30,31]. During natural contexts (i.e., outside of the experimental events), data on these females were collected using focal animal and *ad libitum* sampling [32]; when more than one female was proceptive, observers split up and followed different females independently. Focal samples on proceptive females lasted for 30 min and a minimum of two focal sessions per day was collected. Focal sampling was based on a combination of instantaneous and continuous recording. Specifically, instantaneous recording was used at 1-min intervals to note the occurrence of proceptive displays (see [17] for additional details on data collection and the proceptive signalling repertoire), whereas countinuous recording was used to sample copulations between the focal female and males (see [30] for definitions). *Ad libitum* sampling was used to record the occurrence of all observed proceptive behavior and copulations occurring outside of focal samples during both natural and experimental contexts.

During provisioning experiments, observations were initiated as the study group arrived at the platform site and terminated when all food at the site had been eaten. The identity of all adults and sub-adults feeding from the same platform or in its close proximity (within 1m) was scored instantaneously every 30s by the observers, each of them collecting data at a different platform.

### Determination of female reproductive state

In order to monitor female reproductive status, fecal samples (N = 953) were collected opportunistically from the 12 study females during our two study periods. Urine-free aliquots of fecal samples were collected from identified females within 30 minutes of defecation and stored in polypropylene tubes. Samples were stored in ice bags and moved into a -15°C freezer in the field laboratory where they were stored until extraction (see [33] for details on extraction methods). Fecal extracts were analyzed for concentrations of progesterone metabolites (5α-reduced-20-oxo pregnanes, 5-P-3OH) by enzyme-immunoassay (EIA) as described by Tiddi et al. (2015). The measurement has been shown to provide reliable information on ovarian activity, timing of ovulation and female fertile periods (i.e., peri-ovulatory periods) as well as on gestation in several primate species [34–36], including black capuchin monkeys [17]. The pattern of fecal progesterone metabolites, determined in a previous study [17], were used to assess female reproductive state during the experiments (see Table A in S1 File). Additionally, we verified that events of female proceptivity were hormonally correlated with their fertile period.

### Ethics statement

This study was approved by the Animal Welfare Officer at the German Primate Center (DPZ) and by the Argentine Administration of National Parks (permit number: NEA 158 bis Rnv 5), and adhered to the legal requirements of Argentina.

### Statistical analysis

We measured female mating preference across the two experimental conditions considering, first, a daily measure of each female’s sexual solicitation directed to the alpha male, and, second, a daily measure of each female’s copulation with the alpha male. In the first case, for each observation day in which females showed proceptivity (N = 117), we calculated the proportion of female solicitations to the alpha male from our individual focal samples. To obtain this, we calculated the total number of instantaneous points the focal female was observed soliciting the alpha male and divided it by the total number of points the same female solicited any male in her group to calculate a daily proportion of solicitations directed to the alpha male. Since the proportion of solicitations directed to the alpha male per day was usually 1 or 0 (i.e., either all of the solicitations or none of them were directed to the alpha male during all but 7 days) we dichotomized this variable such that it indicates whether the majority of solicitations was directed to the alpha or not (scored as 1 or 0, respectively, after excluding one day during which exactly half of the solicitations were directed to the alpha). To test whether female mating preference was correlated with the degree of resource control shown by the alpha male (high versus low resource control conditions), we then fitted a Generalized Linear Mixed Model (GLMM) [37] with binomial error structure and logit link function [38]. The categorical response was whether the majority of the solicitations were directed to the alpha male or not (1 or 0). We included experimental condition (high versus low resource control conditions), female rank and the number of cycles until conception as fixed effect predictors. Female identity was included as random effect. Note that we excluded females from the Spot group in this analysis because all solicitations were invariably directed to the alpha male, causing a complete separation problem [39]. To keep type I error rate at the nominal level of 0.05, we included random slopes [40,41] of cycle number and experimental conditions within female. We did not include the correlation among the random slopes and the intercept because it appeared unidentifiable as indicated by an absolute value close to one [42]. To rule out that the model suffered from the inclusion of unidentifiable random slopes, we refitted without any random slopes included.

As a second measure of female mating preference, we calculated female copulation patterns according to two categories: whether on that day the female copulated exclusively with the alpha male (scored as 1), or whether she copulated with any other male in the group (regardless if she also copulated with the alpha male; scored as 0). These patterns of copulations were calculated considering only those days in which copulations were observed (N = 65). We then fitted a second model with the categorical response being female copulation pattern with the alpha male (1 or 0) during a given day. This model was largely identical the solicitations model with the exception that this time we could include data of both groups (and hence also a fixed effect of group), but we could not include a random slope of condition.

Both models were fitted in R (version 3.4.1) [43] using the function glmer of the package lme4 (version 1.1–13) [44]. Prior to fitting the model, we z-transformed rank and number cycles to a mean of zero and a standard deviation of one to achieve comparable estimates and ease fitting the model. Collinearity among the predictors [39] was assessed using the function “vif “of the R package “car” [45], applied to a standard linear model lacking the random effects, and appeared to not be an issue (maximum Variance Inflation Factor: 1.2). In order to assess model stability, and thus to facilitate the interpretability of non-significant results, we compared the estimates derived for the full model with those obtained from bootstrapped subsets of data, obtained by dropping females one at a time. The sample size for the solicitations model was 117 observation days of 9 females (number of days with majority of solicitations directed to the alpha: N = 85); the sample size for the copulations model was a total 65 observation days of 12 females (number days with copulations with the alpha male exclusively: N = 42). Finally, because females in *Sapajus* spp. tend to show a cyclical pattern in their overall interest towards the alpha male that follows the approach of ovulation [16–18], these two models were rerun including only observation days within cycles in which ovulation was precisely pinpointed and adding the day to ovulation as further fixed effect (see [17]).

As an additional analysis, we explored female features (reproductive and social parameters) that may influence the distribution of feeding tolerance by the alpha male. Specifically, we analyzed co-feeding patterns between the alpha male and females during the clumped food condition. By focusing only on trials where the alpha male had full control over food access, we were able to evaluate whether female reproductive states influenced her likelihood to co-feed with this male. Here, we used a multi-level mixed-effect negative binomial regression to determine whether female reproductive state (e.g., cycling, non-cycling and pregnant) had any effect on the likelihood of co-feeding on a platform with the alpha male. Daily counts of instances of co-feeding between females and the alpha male was the response variable, and female reproductive state (three categories) was the predictor variable. Co-feeding counts between the alpha male and females were calculated considering those experimental trials in which the two groups were exposed to high resource control condition (N = 448). The exposure variable was the daily instances of feeding on platforms by the alpha male. Female ID and group ID were entered as random factors. Female dominance rank was an additional fixed effect entered in this model. This negative binomial mixed model regression was run using Stata 13.0 (StatCorp, College Station, TX). Significance values were set at P<0.05 and all reported probabilities were two-tailed.

In order to assess the predictive power of our study, we ran a power analysis informed by effect sizes from previous studies of capuchins under different experimental feeding regimes (specifically, a study which used feeding platforms to test for a difference in capuchin alarm call production between clumped and dispersed conditions [28]) to determine the power of our analysis to detect an effect of this size. From this previous study, we extracted an effect size (r value [46]) of 0.737. Based on a sample size of N = 12 females, we obtained a power of 0.62 for the current analyses using G*Power [47]. Note that because the statistical design was reconceived as t-test in G*Power (to overcome difficulties in conducting such a power analysis with mixed effects models), the obtained predictive power is likely an underestimation of the real power achieved in our GLMMs.

## Results

### Alpha male resource control and female mating preference

**Fig 1:**
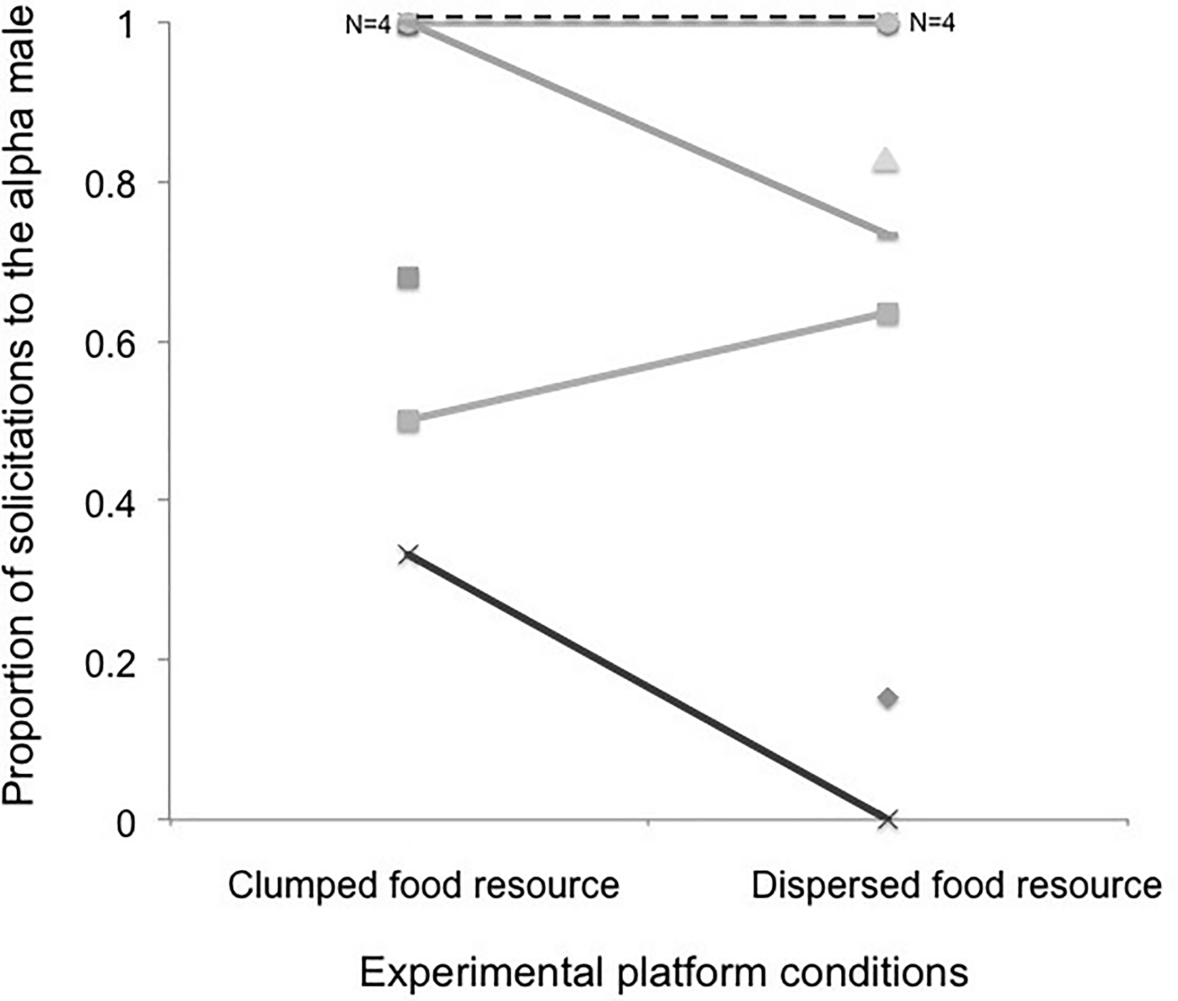
Proportion of solicitations females directed to the alpha males in their group (Mean ± SD) in relation to the experimental platform condition (high versus low resource monopolizability). Each study female is represented by a unique symbol, and each grey scaled or dashed line connects a given female’s values across the two experimental conditions. Symbols without connecting lines correspond to 6 females observed during only one of the two conditions (see “Sampling year” in Table A in S1 File).

**Fig 2:**
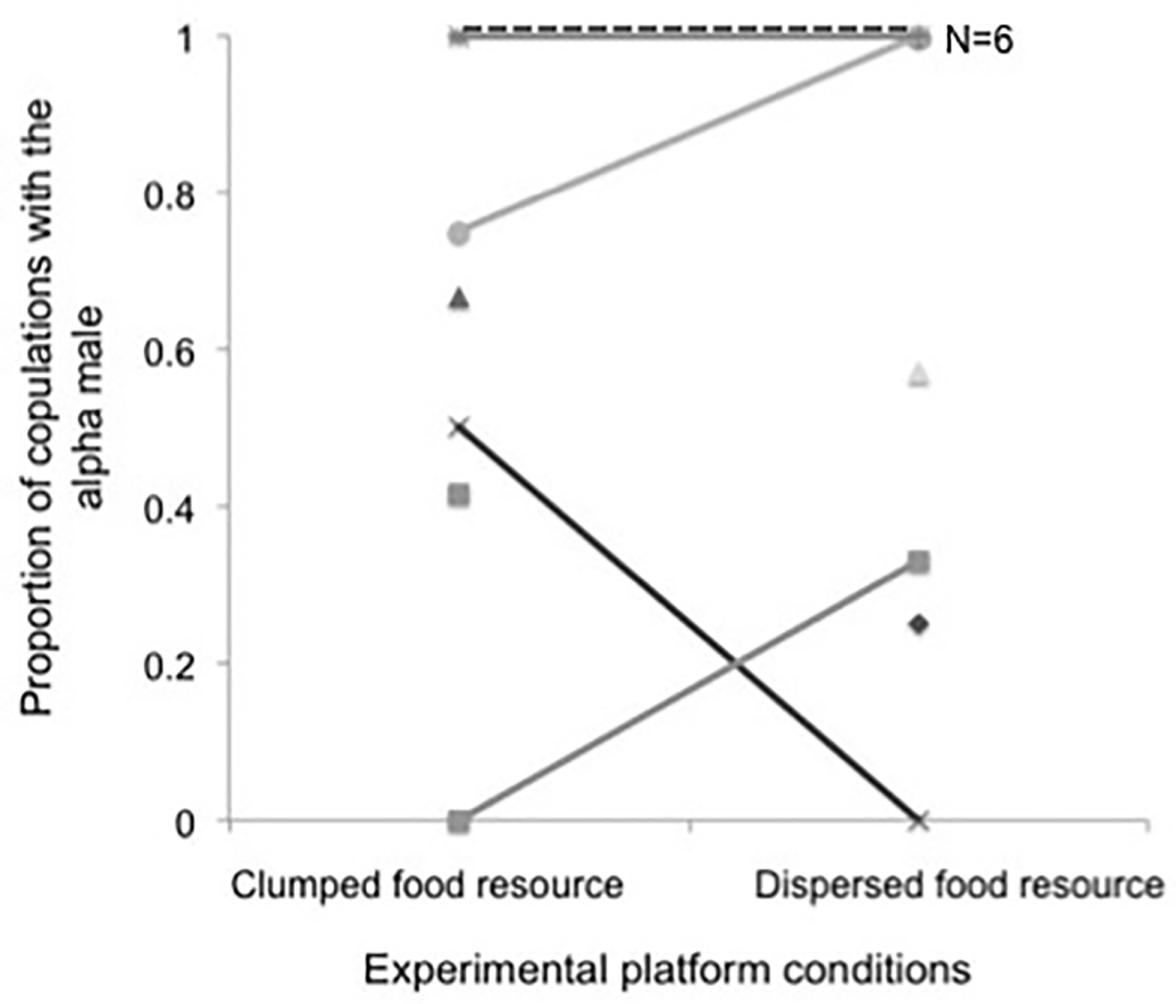
Proportion of copulations females performed with the alpha males in their group (Mean ± SD) in relation to the experimental platform condition (high versus low monopolizability). Each study female is represented by a unique symbol, and each grey scaled or dashed line connects a given female’s values across the two experimental conditions. Symbols without connecting lines correspond to 7 females observed during only one of the two conditions (see “Sampling year” in Table A in S1 File).

Females overall showed a strong and consistent preference for the alpha males (mean ± SD proportion of solicitations: 0.79 ± 0.28 in the high and 0.71± 0.39 in the low resource control condition; mean ± SD proportion of copulations: 0.62± 0.35 in the high and 0.71± 0.40 in the low resource control condition). Neither experimental condition (i.e., low *vs*. high resource control) nor any of the other two predictors (e.g., female rank and cycle number) had a significant effect on whether solicitations were more frequently directed towards the alpha male (Table 1; Fig 1). Female copulation pattern was also not influenced by experimental condition, female rank, or number of cycles until conception (Table 2; Fig 2). Results did not change when considering only observation days within cycles in which ovulation was precisely pinpointed and adding the day to ovulation as further fixed effect (see Tables B and C in S1 File).

**Table 1:** Results of the solicitations GLMM (estimates, SEs, confidence intervals, results of likelihood ratio tests and minimum and maximum of estimates derived when excluding females one at a time).

Variables	Estimate	SE	lower Cl	upper Cl	χ^2^	df	p	min	max
**Intercept**	1.772	0.941	-0.019	4.606			^(1)^	1.200	2.837
**Condition^(2)^**	-0.830	0.873	-3.636	1.086	0.930	1	0.335	-2.140	0.160
**Rank^(3)^**	-0.312	0.694	-1.938	1.223	0.185	1	0.667	-0.993	0.383
**Cycle nr.^(4)^**	0.077	0.524	-1.070	1.385	0.020	1	0.889	-0.382	0.489

N = 117 observation days on 9 proceptive females. Female ID was entered as random factors.

^(1)^ not indicated because of having a limited interpretation.

^(2)^ dummy coded with clumped being the reference category.

^(3)^ z-transformed, mean and standard deviation of the original variable were 4.709 and 2.317, respectively.

^(4)^ z-transformed, mean and standard deviation of the original variable were 2.530 and 1.229, respectively.

**Table 2:** Results of the female copulation pattern GLMM (estimates, SEs, confidence intervals, results of likelihood ratio tests and minimum and maximum of estimates derived when excluding females one at a time).

Variables	Estimate	SE	lower Cl	upper Cl	χ^2^	df	p	min	max
**Intercept**	0.167	0.690	-1.479	1.810			^(1)^	-0.332	0.786
**Condition^(2)^**	0.479	0.806	-1.261	2.438	0.356	1	0.551	-0.024	1.196
**Rank^(3)^**	0.247	0.532	-0.947	1.600	0.222	1	0.638	-0.195	0.878
**Cycle nr.^(4)^**	0.160	0.392	-0.820	1.145	0.167	1	0.683	-0.104	0.566
**Group^(5)^**	2.754	1.470	0.423	20.533	4.238	1	0.040	2.073	18.495

N = 65 observation days on 12 proceptive females mating with group males. Female ID was entered as random factors.

^(1)^ not indicated because of having a limited interpretation

^(2)^ dummy coded with clumped being the reference category

^(3)^ z-transformed, mean and standard deviation of the original variable were 4.315 and 2.324, respectively

^(4)^ z-transformed, mean and standard deviation of the original variable were 2.338 and 1.189, respectively

^(5)^ dummy coded with group MAC being the reference category; the very large instability of the estimate (column 'max') was driven by the fact that, in one group (Spot), copulations were monopolized during all but one day, and complete separation occurred when this was excluded.

### Factors influencing the relationship between male resource control and female mating preference

**Fig 3:**
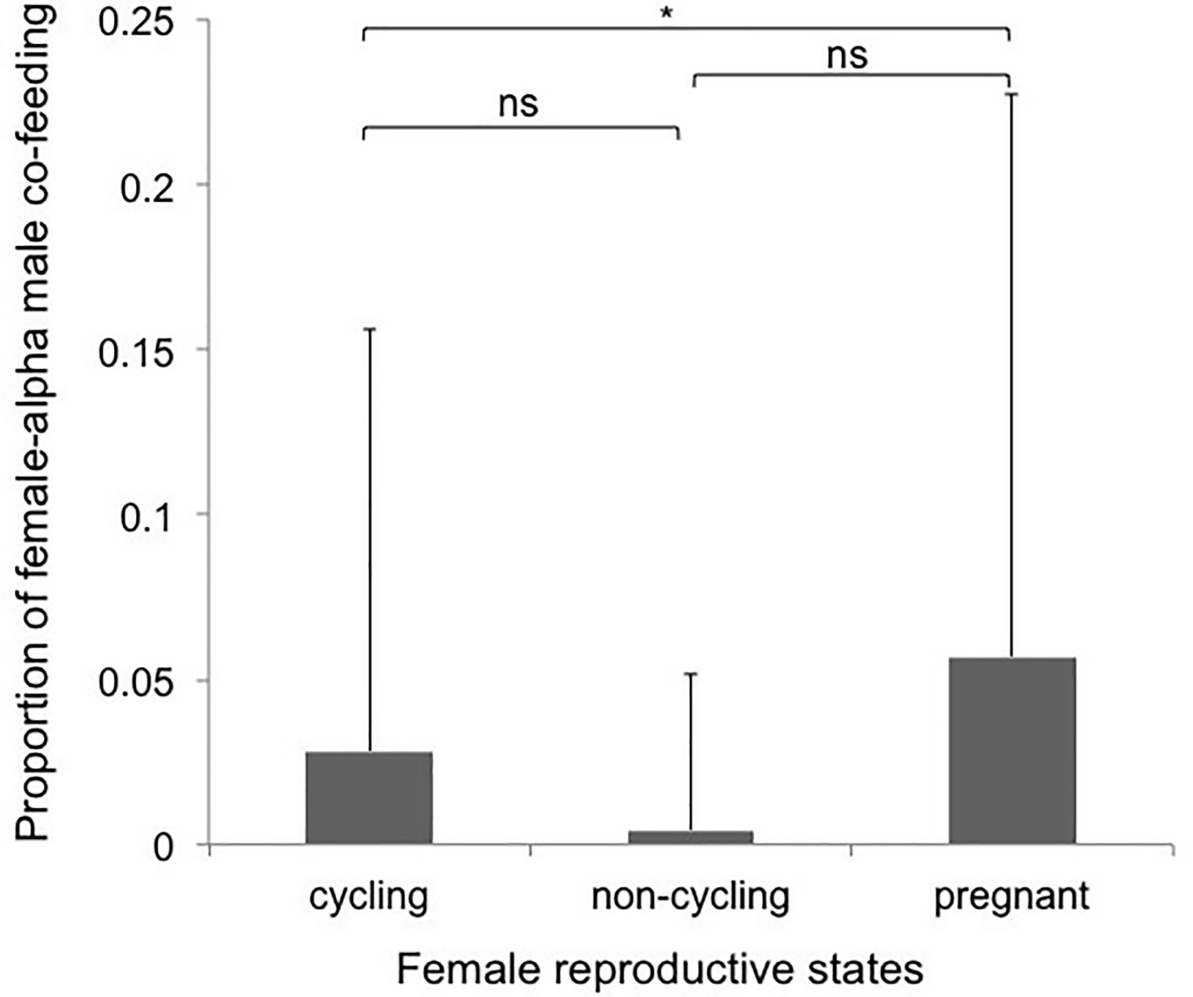
Proportion of co-feeding during platform experiments (Mean ± SD) across the three female reproductive states (cycling, non-cycling, and pregnant). For this analysis we only included data from the high monopolizability condition during the platform experiments.

We conducted 149 trials of the high resource control condition (i.e., one platform per site) for the two groups. Of these, co-feeding between females and the alpha male in each group was observed in only 33 trials. Therefore, females in both groups had little access to high quality food during trials in which the alpha male exercised full control over the resource. However, cycling females co-fed with alpha males significantly more than non-cycling females (Table 3; Fig 3).

**Table 3:** Results of a multi-level mixed-effect negative binomial regression testing instances of co-feeding between females and alpha male as the response variable, female reproductive states as categorical explanatory factors, and controlling for female dominance rank. Instances of alpha male presence on platforms were used as the exposure variable.

Variables	Estimate	SE	z	P	Lower CI	Upper CI
**Cycling/ non-cycling**	-2.677	1.325	-2.02	0.043	-5.274	-0.079
**Cycling/pregnant**	-1.236	0.801	-1.55	0.121	-2.799	0.327
**Non-cycling/pregnant**	1.441	1.567	0.92	0.358	-1.631	4.513
**Female rank**	-0.425	0.243	-1.75	0.081	-0.901	0.051

N = 448 experimental events during the clumped condition (Macuco group: 36 trials, each of them with 7 female-alpha-male co-feeding values; Spot group: 49 trials, each of them with 4 female-alpha-male co-feeding dyadic values). Subject ID and group ID were entered as random factors.

## Discussion

Our study investigated the potential link between ecological factors, specifically the distribution of food resources, and mating strategies in a wild population of robust capuchin monkeys, a genus whose mating system has been suggested to resemble a resource defense polygyny [16,19,20]. Although corroborating previous studies indicating that alpha males are the preferred mates of female robust capuchins, our results indicated that the degree of food resource control exerted by the top-ranking males in their groups was not related to female sexual preferences in this species. Specifically, we showed that when experimentally reducing the potential for alpha males to control access to resources (3-fold difference in the amount of food contestable by a single individual between the clumped and dispersed conditions, and with additional males able to monopolize the same amount of food as the alpha male in the latter condition), the proportion of female sexual solicitations and copulations with the alpha male did not significantly differ relative to conditions in which such control was increased. This suggests that within-group male resource defense might not be a mating strategy in this species, as females still preferred top-ranking males as mating partners even when their ability to control access to food was similar to that of other males. It is important to note, however, that the width of confidence intervals suggests that there may be some uncertainty in the effect of the experimental condition on the responses. Although this may be the consequence of small sample size, previous field studies on the same wild population under similar experimental conditions and number of subjects did report significant results [17,48]. A lack of hypothesis support is also in line with behavioral observations in natural conditions on other populations of black capuchins showing a degree of fission-fusion dynamics [49,50] and thus variable control over food resources by the alpha male (see [51]). In this case, females spending most of their time in a party without the alpha male did not solicit other males and still showed a high preference towards the alpha male when the group reunited [52].

The absence of a change in female sexual preference in our species may potentially be explained as phylogenetic effects on this behaviour. Specifically, the tendency for robust capuchin females to show a strong preference for alpha males [16,19,20] may be a relatively inflexible strategy that has been selected in the past because only alpha males, but not lower ranking males, exert a high degree of control over access to food resources [19]. If so, female robust capuchins may lack the behavioural plasticity to respond to novel, short-term changes in local ecological conditions (like those experimentally induced in the current study) with changes in mate preference. Indeed, previous studies have suggested that phylogenetic history may have constrained sexual behaviour of robust capuchin monkeys in other ways at both the species and genus level [31,53]. More broadly, phylogenetic analyses show relatively few changes from primitive to derived mating systems occurring in the course of primate evolution [54], suggesting that mating behaviour may in fact be highly constrained (see also [55]).

Alternatively, it is possible that the females do indeed have some flexibility regarding their degree of promiscuity, but that the temporal window chosen in our study (i.e., one 3.5-month mating season for each experimental condition) was not long enough to induce any change in female sexual behavior, and thus to drive a shift towards greater promiscuity. This aspect of the experimental design, however, seems appropriate given previous observations of robust capuchin females in both captive and field contexts which have described several examples of situation-dependent receptivity associated with the appearance of new males in the group or change in male dominance ranks (reviewed in [18]). This might suggest that, in our study subjects, changes in female sexual behavior may be more strongly triggered by changes in the social context (e.g., periods of social stability versus periods of instability) rather than the feeding context. Indeed, changes in alpha male status are associated with increased risk of infanticide by group males [56], and this may play an important role in shaping the degree of female behavioral flexibility in terms of mate preference during periods of instability. Specifically, females may show less preference towards the alpha male during periods of instability in male dominance hierarchies in order to sow paternity confusion and reduce the possibility of infanticide by potential new alpha males [57]. During the current study, social rank among males was stable and this, rather than a male’s current ability to monopolize food patches, may be the more salient factor for females when making mating decisions (see also [20]).

Our results provide new insights into the feeding ecology of the species by indicating that females in both study groups experienced little tolerance from the alpha males in the form of experimental co-feeding on high-quality clumped food resources. Such tolerance was partially dependent upon female reproductive state, with cycling females receiving significantly more tolerance than non-cycling females (although it’s not clear if this is due to a more general difference in overall feeding rates between cycling and non-cycling females). A previous study on the same population showed that the sex of the subordinate significantly influenced the interchange between grooming and tolerance while feeding at the platforms, with sub-adult and juvenile males being more tolerated by dominant individuals (regardless of their sex), than were females [58]. As such, direct benefits expressed as increased access to resources for females when food is highly contestable may be less important than initially predicted [16,19]. It still remains possible, however, that females prefer to mate with males that best control access to resources because these males will be able to provide tolerance to their shared offspring in feeding contexts [19,20]; paternity data and detailed observations of alpha male-infant interactions during feeding are needed in order to confirm this possibility.

Finally, female mating preference may be driven by aspects related to the alpha status in male robust capuchins that go beyond feeding contexts. A previous study on the same population has shown that alpha males represent the most socially integrated males in the group, and females with high dominance rank and high centrality in both proximity and grooming networks showed stronger relationships with the alpha male [59]. It is possible that in a social context females may gain some benefits by associating preferentially with the alpha male given his potential role as a policing individual (see [60]), or in counterattacking predators [19,61].

In conclusion, our findings indicate that, in our study population, female preference was not contingent upon the alpha male ability to monopolize food resources during the mating season, contrasting with previous suggestions that robust capuchins show a resource defense polygyny mating system [16,19]. Future studies should attempt to investigate two interesting yet untested aspects of female mating preference in this species. First, female mating preference should be investigated by considering the role of alpha males in additional contexts, such as social support in agonistic contexts and anti-predator behavior. This might help to fully understand decision-making processes behind female mating preference in this species. Second, because lactating female capuchins increased their energy intake by increasing their feeding rate in fruit patches [62], it remains possible that lactating females may depend more strongly than pregnant or cycling females from tolerance during feeding provided by alpha males. Such studies may clarify which features of alpha males drive female robust capuchins to prefer to mate with these over lower-ranking males.

## Supporting information

S1 File **Table A.** Group membership, dominance rank and reproductive state of the black capuchin females in Iguazú**. Table B.** Results of Generalized Linear Mixed Model with binomial error structure and logit link function testing the male-resource control hypothesis for capuchin female mating preferences. **Table C.** Results of GLMM with binomial error structure and logit link function testing the male-resource control hypothesis for capuchin female mating preferences.
(DOCX)
