# Supplementary material for: Male resource defense mating system in primates? An experimental test in wild capuchin monkeys
Source: PLoS One. 2018 May 22;13(5):e0197020. doi: 10.1371/journal.pone.0197020 (PMC5963770; doi:10.1371/journal.pone.0197020)
Supplement: S1 File — Table A. Group membership, dominance rank and reproductive state of the black capuchin females in Iguazú. Table B. Results of Generalized Linear Mixed Model with binomial error structure and logit link function testing the male-resource control hypothesis for capuchin female mating preferences. Table C. Results of GLMM with binomial error structure and logit link function testing the male-resource control hypothesis for capuchin female mating preferences. (DOCX) [file pone.0197020.s001.docx]

**Resource defense polygyny in capuchin monkeys? Female response to experimental manipulation of male resource control**

Barbara Tiddi, Michael Heistermann, Martin K. Fahy, Brandon C. Wheeler

**S1 File. Table A.** Group membership, dominance rank and reproductive state of the black capuchin females in Iguazú

| **Female ID** | **Group** | **Sampling year** | **Age class***** | **Dominance rank** | **Reproductive state** (in 2012)* | **Reproductive state** (in 2013)* |
| --- | --- | --- | --- | --- | --- | --- |
| Cordelia | Macuco | 2013 | SUBAD | 7.5 | Non-cycling 21 | Cycling 38 |
| Estela | Macuco | 2012/13 | AD | 2 | Cycling 52 | Cycling 33 |
| Ingrid | Macuco | 2012/13 | SUBAD | 4 | Cycling 49 | Cycling 40 |
| Maw | Macuco | 2012 | SUBAD | 5.5 | Cycling 50 | Non-cycling 35 |
| Ofelia | Macuco | 2012/13 | SUBAD | 5.5 | Cycling 49 | Cycling 36 |
| Sol | Macuco | 2012 | AD | 7.5 | Cycling 41 | Not sampled** |
| Thelma | Macuco | 2012/13 | AD | 1 | Cycling 57 | Cycling 39 |
| Yoli | Macuco | 2012 | AD | 3 | Cycling 59 | Non-cycling 39 |
| Bianca | Spot | 2013 | AD | 1 | Pregnant 39 | Cycling 48 |
| Daniela | Spot | 2012 | SUBAD | 3 | Cycling 27 | Non-cycling 31 |
| Eva | Spot | 2012/13 | AD | 2 | Cycling 35 | Cycling 51 |
| Josefa | Spot | 2012/13 | AD | 4 | Cycling 33 | Cycling 35 |

***** Female reproductive state at the beginning of each data collection period

****** Insufficient number of samples

***SUBAD: Sub-adult females; AD: adult females

**S1 File. Table B**. Results of Generalized Linear Mixed Model with binomial error structure and logit link function testing the male-resource control hypothesis for capuchin female mating preferences using a categorical measure of female solicitation to the alpha male as the response variable (1 for the majority of solicitations directed to the alpha male and 0 for the opposite situation), platform condition (low versus high resource control) as the categorical explanatory factor, and controlling for the effects of female dominance rank, cycle to ovulation and the day of sampling relative to subjects’ ovulation. Data points included only those cycles in which ovulation was pinpointed.

| Variables | Estimate | SE | z | P |  |  | Lower CI |  |  |  | | Upper CI | |  | |  | |  |
| --- | --- | --- | --- | --- | --- | --- | --- | --- | --- | --- | --- | --- | --- | --- | --- | --- | --- | --- |
| Intercept | 18.628 | 30.998 | 0.60 | 0.548 |  |  | -42.126 |  |  | |  | | 79.382 | |  | |  |  |
| Platform condition | -6.238 | 8.392 | -0.74 | 0.457 |  |  | -22.688 |  |  |  | | 10.211 | |  | |  | |  |
| Female rank | 0.216 | 1.190 | 0.18 | 0.856 |  |  | -2.115 |  |  |  | | 2.550 | |  | |  | |  |
| Cycle nr. | -0.170 | 0.464 | -0.37 | 0.714 |  |  | -1.079 |  |  |  | | 0.739 | |  | |  | |  |
| Day to ovulation | 0.046 | 0.215 | 0.22 | 0.829 |  |  | -0.376 |  |  |  | | 0.469 | |  | |  | |  |

N=62 observation days on 8 proceptive females from Macuco group. Subject ID was entered as random factors.

**S1 File. Table C**. Results of GLMM with binomial error structure and logit link function testing the male-resource control hypothesis for capuchin female mating preferences using categorical measures of copulations with the alpha male as the response variable, platform condition (low versus high resource control) as the categorical explanatory factor, and controlling for the effects of female dominance rank, cycle to ovulation and the day of sampling relative to subjects’ ovulation. Analysis was limited to cycles in which ovulation was pinpointed.

| Variables | Estimate | SE | z | P |  |  | Lower CI |  |  |  | | Upper CI | |  | |  | |  |
| --- | --- | --- | --- | --- | --- | --- | --- | --- | --- | --- | --- | --- | --- | --- | --- | --- | --- | --- |
| Intercept | -23.620 | 40.075 | -0.59 | 0.556 |  |  | -102.165 |  |  | |  | | 54.925 | |  | |  |  |
| Platform condition | 3.434 | 10.062 | 0.34 | 0.733 |  |  | -16.287 |  |  |  | | 23.154 | |  | |  | |  |
| Female rank | 0.115 | 1.582 | 0.07 | 0.942 |  |  | -2.986 |  |  |  | | 3.216 | |  | |  | |  |
| Cycle nr. | 0.333 | 0.620 | 0.54 | 0.591 |  |  | -0.882 |  |  |  | | 1.548 | |  | |  | |  |
| Day to ovulation | -0.473 | 0.361 | -1.31 | 0.189 |  |  | -1.180 |  |  |  | | 0.233 | |  | |  | |  |

N=35 observation days on 9 proceptive females mating with group males. Subject ID was entered as random factors.
